# Effects of Spaceflight on Musculoskeletal Health: A Systematic Review and Meta-analysis, Considerations for Interplanetary Travel

**DOI:** 10.1007/s40279-021-01496-9

**Published:** 2021-06-11

**Authors:** Paul Comfort, John. J. McMahon, Paul. A. Jones, Matthew Cuthbert, Kristina Kendall, Jason. P. Lake, G. Gregory Haff

**Affiliations:** 1 Human Performance Laboratory, Directorate of Psychology and Sport, University of Salford, Salford, GM UK; 2 School of Medical and Health Sciences, Edith Cowan University, Joondalup, WA Australia; 3 Institute for Sport, Physical Activity and Leisure, Carnegie School of Sport, Leeds Beckett University, Leeds, UK; 4 Technical Directorate Division, The FA Group, St George’s Park, Burton-Upon-Trent, Staffordshire, UK; 5 Chichester Institute of Sport, University of Chichester, Chichester, UK

## Abstract

**Background:**

If interplanetary travel is to be successful over the coming decades, it is essential that countermeasures to minimize deterioration of the musculoskeletal system are as effective as possible, given the increased duration of spaceflight associated with such missions. The aim of this review, therefore, is to determine the magnitude of deconditioning of the musculoskeletal system during prolonged spaceflight and recommend possible methods to enhance the existing countermeasures.

**Methods:**

A literature search was conducted using PubMed, Ovid and Scopus databases. 5541 studies were identified prior to the removal of duplicates and the application of the following inclusion criteria: (1) group means and standard deviations for pre- and post-spaceflight for measures of strength, muscle mass or bone density were reported (or provided by the corresponding author when requested via e-mail), (2) exercise-based countermeasures were included, (3) the population of the studies were human, (4) muscle function was assessed and (5) spaceflight rather than simulated spaceflight was used. The methodological quality of the included studies was evaluated using a modified Physiotherapy Evidence Database (PEDro) scale for quality, with publication bias assessed using a failsafe N (Rosenthal method), and consistency of studies analysed using *I*^2^ as a test of heterogeneity. Secondary analysis of studies included Hedges’ g effect sizes, and between-study differences were estimated using a random-effects model.

**Results:**

A total of 11 studies were included in the meta-analyses. Heterogeneity of the completed meta-analyses was conducted revealing homogeneity for bone mineral density (BMD) and spinal muscle size (Tau^2^ < 0.001; *I*^2^ = 0.00%, *p* > 0.05), although a high level of heterogeneity was noted for lower body force production (Tau^2^ = 1.546; *I*^2^ = 76.03%, *p* < 0.001) and lower body muscle mass (Tau^2^ = 1.386; *I*^2^ = 74.38%, *p* < 0.001). The estimated variance (≤ -0.306) for each of the meta-analyses was significant (*p* ≤ 0.033), for BMD (− 0.48 to − 0.53, *p* < 0.001), lower body force production (− 1.75, *p* < 0.001) and lower body muscle size (− 1.98, *p* < 0.001). Spaceflight results in small reductions in BMD of the femur (Hedges *g* = − 0.49 [− 0.69 to – 0.28]), trochanter (Hedges *g* = − 0.53 [− 0.77 to – 0.29]), and lumbo-pelvic region (Hedges *g* = − 0.48 [− 0.73 to – 0.23]), but large decreases in lower limb force production (Hedges *g* = − 1.75 [− 2.50 to – 0.99]) and lower limb muscle size (Hedges *g* = − 1.98 [− 2.72 to – 1.23]).

**Conclusions:**

Current exercise countermeasures result in small reductions in BMD during long-duration spaceflight. In contrast, such exercise protocols do not alleviate the reductions in muscle function or muscle size, which may be attributable to the low to moderate loads reported by crewmembers and the interference effect associated with concurrent training. It is recommended that higher-load resistance exercise and the use of high-intensity interval training should be investigated, to determine if such modifications to the reported training practices result in more effective countermeasures to the deleterious effect of long-duration spaceflight on the muscular system.

## Key Points

**Table:** 

Existing exercise countermeasures, during long-duration spaceflight, are insufficient in eliminating the deleterious effects of microgravity (µG) on lower body muscle function and muscle mass
Existing concurrent training (resistance training and moderate-intensity aerobic training in the same session) practices have the potential to result in an interference effect exacerbating the effect of µG on the muscular system
The reported loads used by astronauts during resistive exercise are generally insufficient for the maintenance of muscle function during prolonged spaceflight
It is imperative that such decreases in muscle mass and function are resolved for safe interplanetary travel

## Background

It has been well documented that microgravity (µG) associated with spaceflight, especially prolonged spaceflight, results in significant deconditioning of the musculoskeletal system [1–4] and can be exacerbated due to a negative energy balance [1, 5–10]. This deconditioning response has been reported to be progressive, increasing as the mission duration is extended [3, 4, 11]. Therefore, it is imperative that the deterioration of the muscular and skeletal systems is more effectively addressed via appropriate countermeasures, if proposed human interplanetary travel is to be successful.

Assessment of skeletal changes, via X-ray photodensitometry [12], regional bone mineral density (BMD) [13, 14] and calcium balance [15, 16], has been implemented since the Gemini and Apollo missions (1965–1972) [3]. During these short-duration missions (e.g., 12.6 days for Apollo 17), there were no changes in the BMD of the wrists (non-weight bearing), but losses of 5–6% in the calcanei (weight bearing) [3, 13], with greater losses (4.5–7.9%) observed during the longer (29–84 days) Skylab missions (1973–1974) [3, 17]. In comparison, the normal rate of decline in the BMD of the femoral neck and total hip on Earth is reported to be 3.2% (95% CI 1.7–4.7%) over a 5-year period, in men aged 35–65 years [18]. During the Souyez and Mir missions, cosmonauts demonstrated greater reductions in BMD associated with the increased duration of the mission; for example, during Salyut-6 (75–184 days) crew members demonstrated losses in calcaneal bone density of up to 19.8% [11]. During this early period of spaceflight, with no exercise countermeasures incorporated to minimize reductions in total BMD, losses were calculated at ~ 0.5% per month [17, 19], approximately 10 times faster than terrestrial rates of BMD decline [18]. As already noted, reductions in BMD are site specific [3, 19, 20], with LeBlanc et al. [19] reporting that non-weightbearing bones in the arms demonstrate very low rates of decline (0.04 ± 0.88%/month) compared to more rapid declines in weightbearing structures, such as the trochanter (1.56 ± 0.99% / month).

As restoration of BMD is estimated to be 5–6 × slower than the rate of loss [3], minimizing its deterioration during spaceflight is essential, especially for repeated long-duration missions [3, 20], and if future mission lengths increase to accommodate interplanetary travel. While not specific to spaceflight, resistance exercise has been reported to be the most effective mode of activity to prevent or minimize reductions in BMD associated with aging and inactivity [21]. This appears to be enhanced when combined with impact activities such as walking and running [22], highlighting the importance of appropriate exercise countermeasures, which may reduce or eliminate the deleterious effect of µG on the musculoskeletal system.

The first observations of reductions in muscle mass in response to spaceflight were made > 40 years ago, since the Gemini, Apollo, Soyuz and Skylab missions [23]. During three Skylab missions, decreases in total body mass of 2.7 ± 0.3 kg were reported, with > 50% attributed to decreases in lean mass (1.5 ± 0.3 kg) [24]; with such changes partly attributed to an ~ 1000 kcal^.^d^−1^ deficit [7]. Reductions in muscle mass result in impaired muscle function, especially in relation to force production [25–29], which could compromise the capacity of crew members to complete common mission-related tasks. Kozlovskaya et al. [25] reported greater reductions in isokinetic torque at low angular velocities (60°^.^s^−1^) (indicative of changes in maximal force production), compared to higher angular velocities (180°^.^s^−1^) (indicative of changes in power output). Similarly, Antonutto et al. [26] reported large reductions in *explosive* power after 31 days (33%) and 180 days (55%) of spaceflight, with a 25% reduction in cycling power. These decreases in power were substantially greater than the decreases in lean mass of the legs (9–13%) reported during the same missions [30], as would be expected due to neurological (e.g., decreased neural drive) and architectural adaptations (e.g., decreased fascicle length and increased pennation angle) [31, 32]. Greater decreases in cross sectional area, force production and power output have previously been reported in fast twitch fibers compared to slow twitch fibers after short-duration spaceflight [27, 29]. LeBlanc et al. [19] reported that muscle mass is lost at ~ 0.57% per month, although, similar to changes in BMD, this appears to be regional, with minimal reductions in arm muscle mass (0.00 ± 0.77%/month) but more rapid reductions in the legs (1.00 ± 0.73%/month). During 16–28 week missions, reductions in BMD (3.4%) mirrored the reductions in lean mass (3.5%) [19]. In simulated µG, decreases in muscle mass appear to plateau at ~ 70% of baseline values after ~ 270 days [33], along with associated decreases in force production and power output during spaceflight [34, 35].

Changes in neural drive, associated with µG, have been suggested to be the main determinant of reductions in strength and power, with recommendations for the performance of explosive exercise during spaceflight to offset such losses [33], although it is unlikely that such exercise would be optimal in the prevention of atrophy or reductions in strength. In 2010, Narici and di Boer [4] suggested that irrespective of in-flight countermeasures, loss of lower limb muscle mass could be as high as 24% over 197 days, with an increased duration associated with increased atrophy, although this rate of atrophy may not be linear. In addition, as the reduction in muscular power during prolonged spaceflight is substantially greater than the reductions in lean mass, changes in motor unit recruitment patterns and electromechanical efficiency, associated with weightlessness, have been suggested to explain much of the reduction in power [4, 33]. Based on the limited data from Skylab and Mir crewmembers, Lane et al. [36] previously suggested that despite sufficient energy and protein intake combined with exercises, such countermeasures were insufficient to prevent loss of muscle mass; however, resistance exercise was limited at this point (e.g., isokinetic device, spring and elastic resistive devices, each offering limited resistance) [37]. More recently, Fitts et al. [35] recommended a combination of isometric and isotonic exercises as an appropriate exercise countermeasure to decreases in muscle mass and strength, although this was based primarily on human bedrest and animal studies. In recent years, space agency’s approaches to counter musculoskeletal deconditioning associated with µG have improved with the introduction of the interim resistive exercise device (iRED) to the ISS (2000–2009), and more recently (2009 to present) the advanced resistive exercise device (ARED), with the latter permitting increased and more consistent loads during exercise [38, 39]. Similarly, later in 2009, the T2 treadmill was installed, to replace the treadmill with vibration isolation and stabilization system (TVIS), which enables a higher running speed (12 mph vs. 10 mph) [39, 40]. The associated exercise protocols for NASA crewmembers, while on the ISS, are provided in Table 1 [38, 40], with the difference between iRED and ARED being the maximal load capacity of each device. While there are some differences in the recommended exercise countermeasures between space agencies, these are generally subtle and would likely result in minimal differences in adaptive responses; with recommendations for exercise 6 days per week, including treadmill and cycle ergometry and resistance exercise using multiple set (2–4) and repetition ranges (6–15), rotating loading across days [38, 40].

**Table 1:** Example exercise recommendations for crewmembers on the International Space Station

	Resistive Exercise Protocol	Treadmill Protocol	Cycle Ergometer Protocol
Type	Resistive Exercise Device (6–15 repetitions, 3–5 sets), exercises include: Squats, Deadlifts, Sumo Deadlift, Romanian Deadlift, Heel Raises, Single Leg Heel Raises, Single Leg Squats, Bent-over Row, Upright Row, Bench Press	Continuous, Interval or Slope	Continuous, Interval or Hill
Load / Intensity	Daily rotations of 6, 8, 15 repetitions for 3–5 sets Based on pre-flight ARED sessions, calculated from 10 RM + 75% body weight to account for lack of body weight in µG	60%, 75%, 85%^a^ alternating daily	60%, 75%, 85% ^a^ alternating daily
Frequency	6 days / week	4–7 days / week	
Progression	3–5% increase / week	Increase speed or duration across the mission Progressive increase in load from ~ 50% body weight up to ~ 80% body weight	
Duration (average)	60 min per session	30 min per session	30 min per session

^a^ % maximum heart rate relative to the crewmembers’ individual capacity (typically 10–30% lower during Phase 1 [first 2–3 weeks])

ARED = Advanced Resistive Exercise Device

These recommendations vary slightly based on the space agency, e.g., NASA, European Space Agency, Japan Aerospace Exploration Agency, Canadian Space Agency

Interim Resistive Exercise Device load capacity ~ 136 kg; Advanced Resistive Exercise Device load capacity ~ 272 kg

Petersen et al. [38] recently reported that crew members on the ISS show little or no change in BMD or aerobic capacity pre- to post-spaceflight, and that decreases in muscular force are becoming progressively smaller due to improvements in the countermeasures used to counteract deconditioning associated with µG. The maintenance of BMD was attributed to regular exercise (6 days/week) including 30 min of cycle ergometry or treadmill running and the use of the ARED. Resistance exercises include the performance of squats, deadlifts and heel raises, alongside appropriate nutrient intake, including vitamin D supplementation (800 IU/d). It is worth noting, however, that not all astronauts maintain their aerobic capacity, in fact only those who exercised at a higher intensity (79 ± 6% of peak heart rate) and accumulated a greater duration at > 70% peak heart rate (76 ± 30 min/week compared to more moderate intensities [68 ± 20% peak heart rate] and a shorter duration at > 70% peak heart rate [63 ± 32 min/week]), maintained their aerobic capacity [41]. Interestingly, these crewmembers were also fitter pre-flight (VO_2peak_ > 40 ml^.^kg^.^min^−1^) compared to crewmembers who demonstrated reduction in aerobic capacity (VO_2peak_ < 40 ml^.^kg^.^min^−1^) [41]. The effect of baseline fitness highlights the importance of appropriate physical conditioning prior to spaceflight; however, Loehr et al. [40] reported that while physical training is planned and provided for ~ 2 years prior to spaceflight, it is usually the first thing to be omitted from the astronaut’s busy schedule. The provision of clear recommendations regarding exercise intensity and duration to minimize maladaptation to prolonged µG is essential, while considering that increased exercise duration at higher relative intensities will further increase energy and nutrient requirements. The daily duration of ~ 2.5 h allocated to exercise (this includes 90–100 min exercise, dressing, set-up of equipment and cleanup) is also likely to affect compliance, especially when other tasks may have to be prioritized [38, 42].

Payne [43] suggested that the increased duration of spaceflight required for interplanetary exploration may result in severe physical disability in astronauts, preventing a safe return to Earth, in line with previous suggestions [10]. More recently, based on the available data, Laurens et al. [10] predicted that a mission to Mars would currently result in serious health implications attributed to ~ 15% loss in body mass, based on a mean weight loss of 2.4% per 100 days µG [44], although this prediction is based on zero gravity, and does not appear to account for the gravity on Mars (0.38G). While Payne [43] explained that the level of physical deterioration during spaceflight is primarily related to the duration of missions, individual responses to µG and the effectiveness of countermeasures and pre-flight status may also affect the resultant magnitude of deterioration astronauts may experience [41]. If interplanetary travel is to be successful over the coming decades, it is essential that such countermeasures are as effective as possible, given the increase in the duration of spaceflight associated with such missions. The aims of this systematic review and meta-analysis were, therefore, to determine the effects of long-duration spaceflight (≥ 30 days [43]) on BMD and muscle function (force production and muscle mass), while evaluating the effects of existing countermeasures to minimize the deterioration of the musculoskeletal system while in µG. It was hypothesized that existing countermeasures would not be sufficient in eliminating the deleterious effects of long-duration spaceflight.

## Methods

### Study Design

This systemic review design was developed in adherence to the guidelines of the Preferred Reporting Items for Systematic Reviews and Meta-analysis (PRISMA). The PRISMA checklist is used as the basis for reporting systematic reviews [45]. The review protocol was not pre-registered for this review.

### Literature Search

A Boolean/phrase search mode was applied using the following keywords: ‘spaceflight OR spaceflight’ AND ‘strength OR atrophy OR bone density’. The keywords were inputted using this format into the following three databases PubMed, Ovid and Scopus. Filters were applied to all databases to include studies that were presented in peer-reviewed academic journal articles. No restrictions were placed upon the age or sex of subjects. The search timeframe was not date restricted and completed on the 30th June 2020.

### Inclusion and Exclusion Criteria

**Fig. 1:**
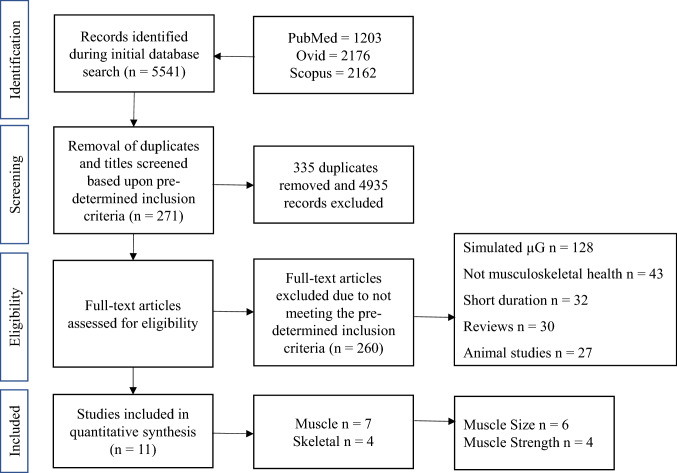
Study selection process. µG = microgravity

The primary focus of this literature search was to identify studies in which the effects of long-duration spaceflight (≥ 30 days [43]) on musculoskeletal health had been investigated, reporting either changes in muscle function (e.g., force production, power), muscle mass (e.g., cross sectional area [CSA], volume, thickness) or BMD. Duplicated studies were removed initially with the remaining studies then being screened, utilizing the subsequent criteria. Research articles were included and eligible within this review provided that (1) group means and standard deviations for pre- and post-spaceflight measures of strength, muscle mass or BMD were reported (or provided by the corresponding author when requested via e-mail), (2) exercise-based countermeasures to mitigate the detrimental effect of µG on muscle force production, muscle mass or BMD were included, (3) the population of the studies were human, (4) muscle function (e.g., strength or power) assessments were included, and finally (5) spaceflight rather than simulated spaceflight / µG was used. Studies which included actual spaceflight were selected as immobilization and bed rest have been reported to be a poor analogue for the study of changes in muscle mass associated with spaceflight, due to differences in activity and energy balance, along with environmental and methodological differences [2, 5, 46, 47]. Studies were excluded if: they were written in a language other than English, were published abstracts (from conference proceedings), did not include means and standard deviations for both pre- and post-spaceflight, if simulated spaceflight/simulated µG were used, if they used animal models (observation of animals in orbit are not ideal models of the effects of spaceflight on human bone [2, 48]), or assessed muscle changes from muscle biopsies, along with any systematic or narrative reviews. In addition, studies including short-duration spaceflight (≤ 30 days [43]) were included in the systematic review but excluded from the meta-analysis. Studies were also excluded from the meta-analyses if data were pooled from multiple missions which included different exercise interventions (e.g., iRED and ARED), as it was not possible to differentiate between the interventions (e.g., Laughlin et al. [49]). A summary of the selection process for the meta-analysis is outlined in Fig. 1.

### Quality and Risk of Bias Assessment

Following the identification of the studies included within this review, the quality and risk of bias were assessed. The methodological quality of the included studies was evaluated using a modified Physiotherapy Evidence Database (PEDro) scale by two authors, with no discrepancies occurring. Given that it is not possible to conceal allocation (point 3) or blind subjects and investigators (points 5–7) in these studies, these points were excluded. A similar approach to the modification of the PEDro scale has been used in previous reviews [50, 51], with ratings adjusted as follows: 5–7 = ‘excellent’; 4 = ‘good’; 3 = ‘moderate’; and 0–2 = ‘poor’. The fail-safe *N* using the Rosenthal method was used to assess publication bias; a fail-safe number of effects calculates the number of un-retrieved null effects that would be needed to diminish the significance of the observed effect and an a priori alpha level of *p* > 0.05; this analysis was carried out using Jamovi [52]. The Cochrane risk of bias assessment tool could not be used to assess risk of bias as no studies were randomized control trials, and blinding of subjects was not possible, but also unlikely to affect the physiological results. In addition, selection bias would always be apparent as only astronauts / cosmonauts could be selected for the studies. An alternative risk of bias assessment was considered using ROBINS-I for observational interventions; however, only two studies [53, 54] included comparative groups which are essential for this method of assessing bias [55].

### Analysis and Interpretation of Results

Six meta-analyses were conducted to compare pre- to post-spaceflight changes in BMD of the femur, trochanter and lumbopelvic region, leg muscle function, leg muscle size and paraspinal muscle size. Means and standard deviations of lower body force production, muscle size and BMD were independently extracted from the included studies for further analysis. Hedge’s *g* effect sizes (ES) and associated 95% confidence intervals (CI) were calculated from the pre- to post-intervention results of each study to provide standardized values whereby the magnitude of differences could be determined and compared across interventions, whilst accounting for differences in sample size. The calculation of Hedges’ *g* was completed using the following formula, (where SD is the standard deviation) [56]:

$$g=\frac{({Mean}_{\mathrm{post}}- {Mean}_{\mathrm{pre}})}{{\mathrm{SD}}_{\mathrm{pooled}}}.$$

The scale for interpretation of ES was proposed by Hopkins [57] as follows: trivial (≤ 0.20), small (0.20–0.59), moderate (0.60–1.19), large (1.20–1.99), or very large (≥ 2.00).

An estimation for between-study variance was calculated using a random-effects model, with associated Z-value, *p*-value and 95% CI; absolute heterogeneity was assessed using Tau^2^ estimated using the restricted maximum likelihood method. Finally, a test for relative heterogeneity (*I*^*2*^) was used to quantify the inconsistency of effects, using a scale of low (< 25%), moderate (25–75%) and high (≥ 75%) [58, 59] with an a priori alpha level of *p* < 0.05. Due to the variance in mission durations of long-duration spaceflight, within individual studies, it was not feasible to include duration as a moderator within the analyses, even though musculoskeletal deterioration has been shown to progress with an increase in mission duration [3, 4, 11]. In addition, due to the low number of studies using different exercise protocols, it was not feasible to use exercise protocol as a moderator.

## Results

### Search Results

Five thousand, five hundred and forty-one studies were identified within the three databases highlighted in Sect. 2.2. Of the total studies identified, 335 articles were duplicates and, therefore, removed first, following the application of the predetermined inclusion/exclusion criteria to both titles and abstracts of the identified studies. Following further inspection of the full text, if required, a total of 11 studies remained for further analysis (Fig. 1). Results include *n* = 138 astronauts/cosmonauts across the 11 studies, ranging from *n* = 4 to *n* = 25 per study.

### Systematic Review and Meta-analyses Findings

**Fig. 2:**
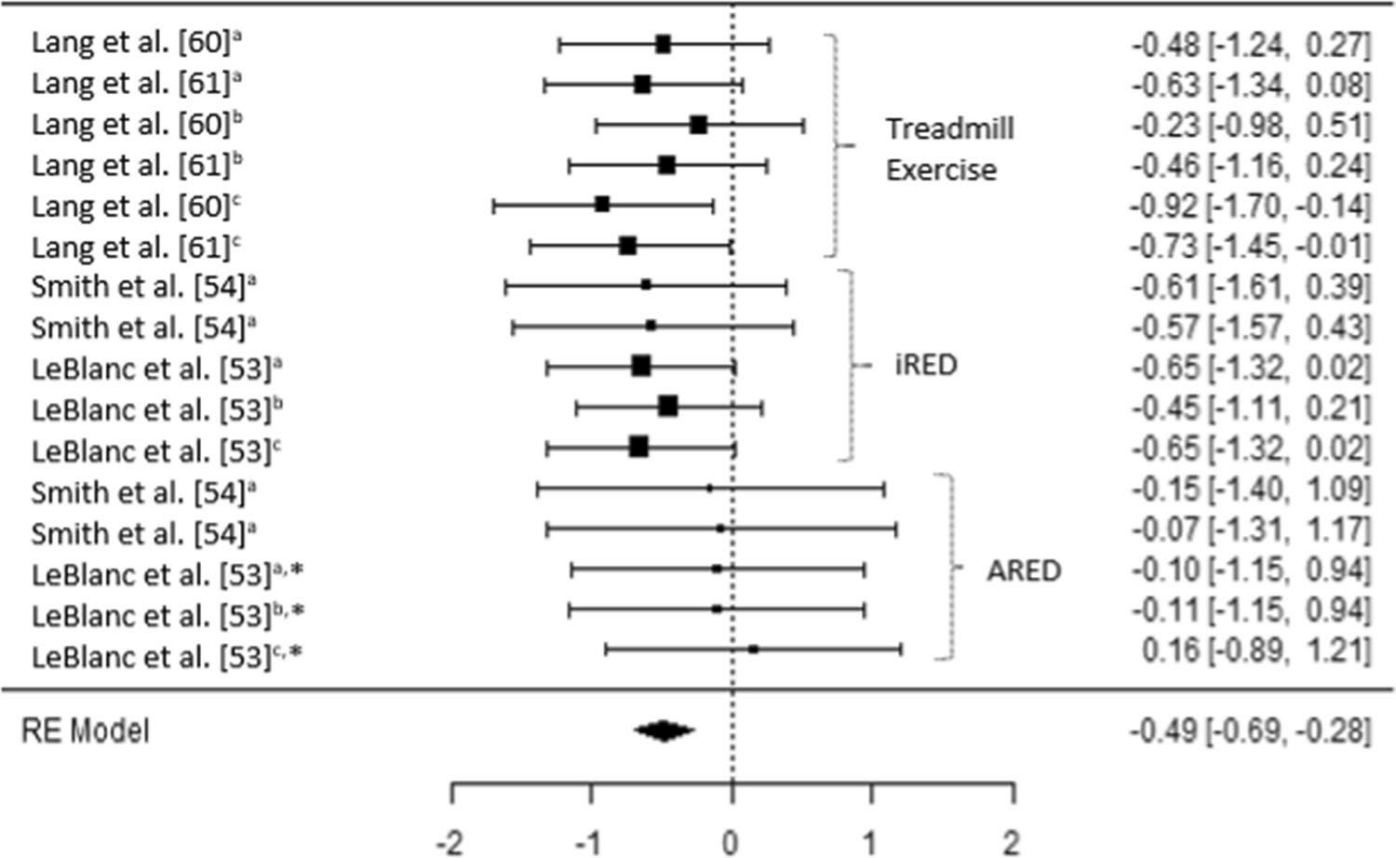
A comparison of changes (effect sizes and 95% confidence intervals) in femoral bone mineral density pre- to post-spaceflight. ^a^ = Integral; ^b^ = cortical; ^c^ = trabecular; * = bisphosphonates administered; iRED = interim resistive exercise device; ARED = advanced resistive exercise device. Values represent Hedge’s *g* effect size and 95% confidence intervals. Negative values (< 0.00) highlight a negative effect

**Fig. 3:**
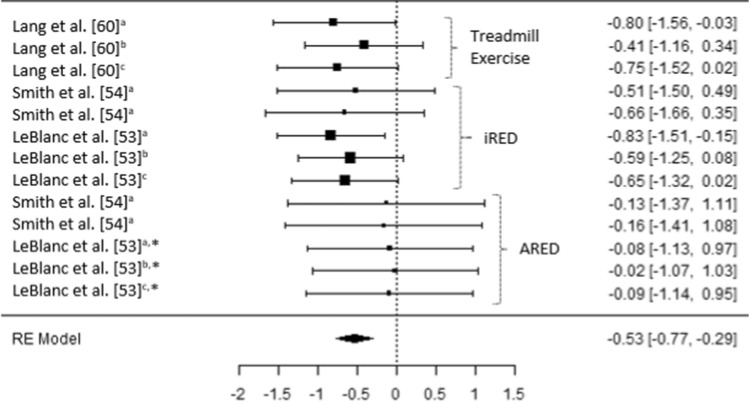
A comparison of changes (effect sizes and 95% confidence intervals) in trochanter bone mineral density pre- to post-spaceflight. ^a^ = Integral; ^b^ = cortical; ^c^ = trabecular; * = bisphosphonates administered; iRED = interim resistive exercise device; ARED = advanced resistive exercise device. Values represent Hedge’s *g* effect size and 95% confidence intervals. Negative values (< 0.00) highlight a negative effect

**Fig. 4:**
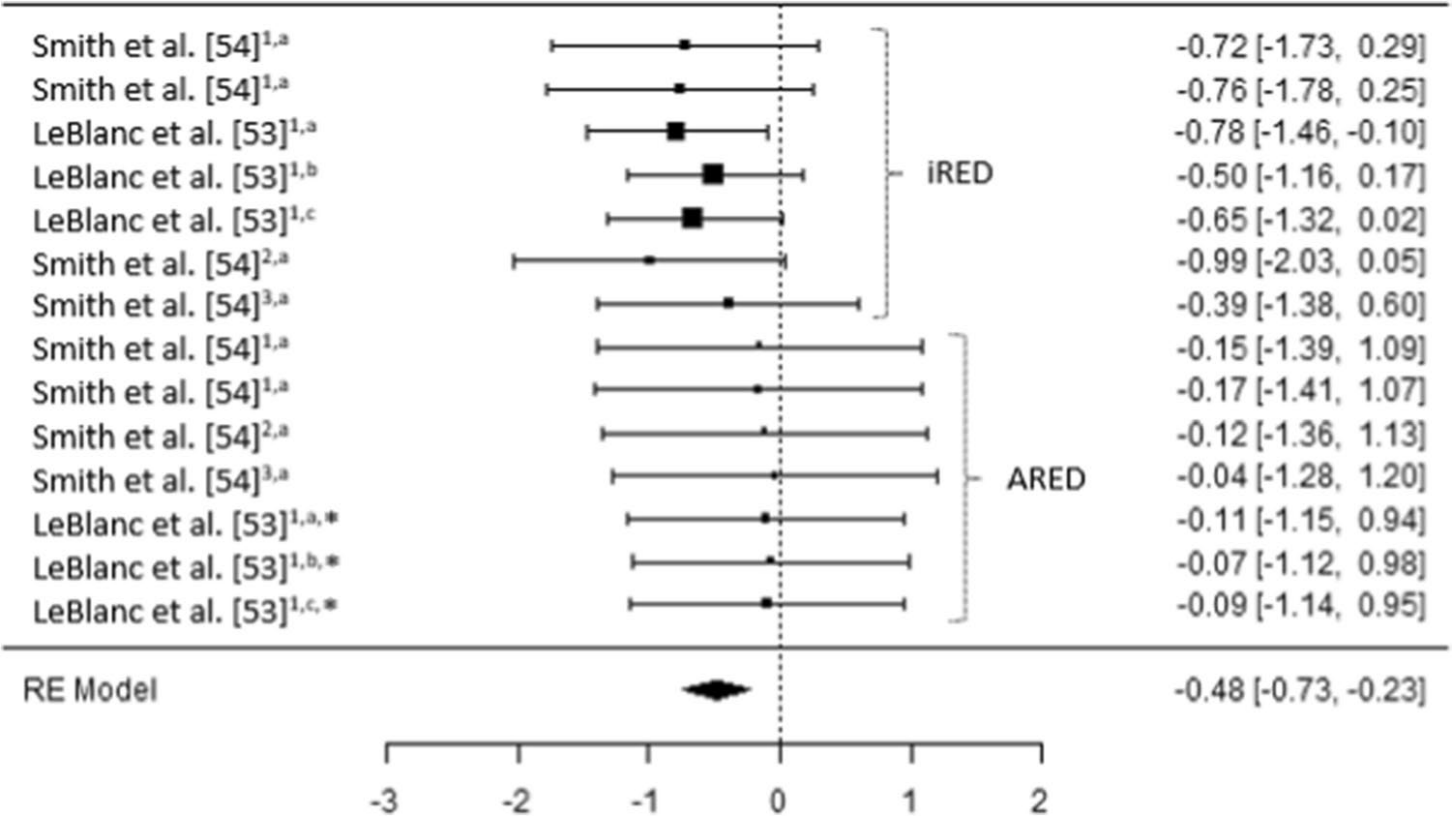
A comparison of changes (effect sizes and 95% confidence intervals) in hip^1^, pelvis^2^ and lumbar spine^3^ bone mineral density pre- to post-spaceflight. ^a^ = Integral; ^b^ = cortical; ^c^ = trabecular; * = bisphosphonates administered; iRED = interim resistive exercise device; ARED = advanced resistive exercise device. Values represent Hedge’s *g* effect size and 95% confidence intervals. Negative values (< 0.00) highlight a negative effect

**Fig. 5:**
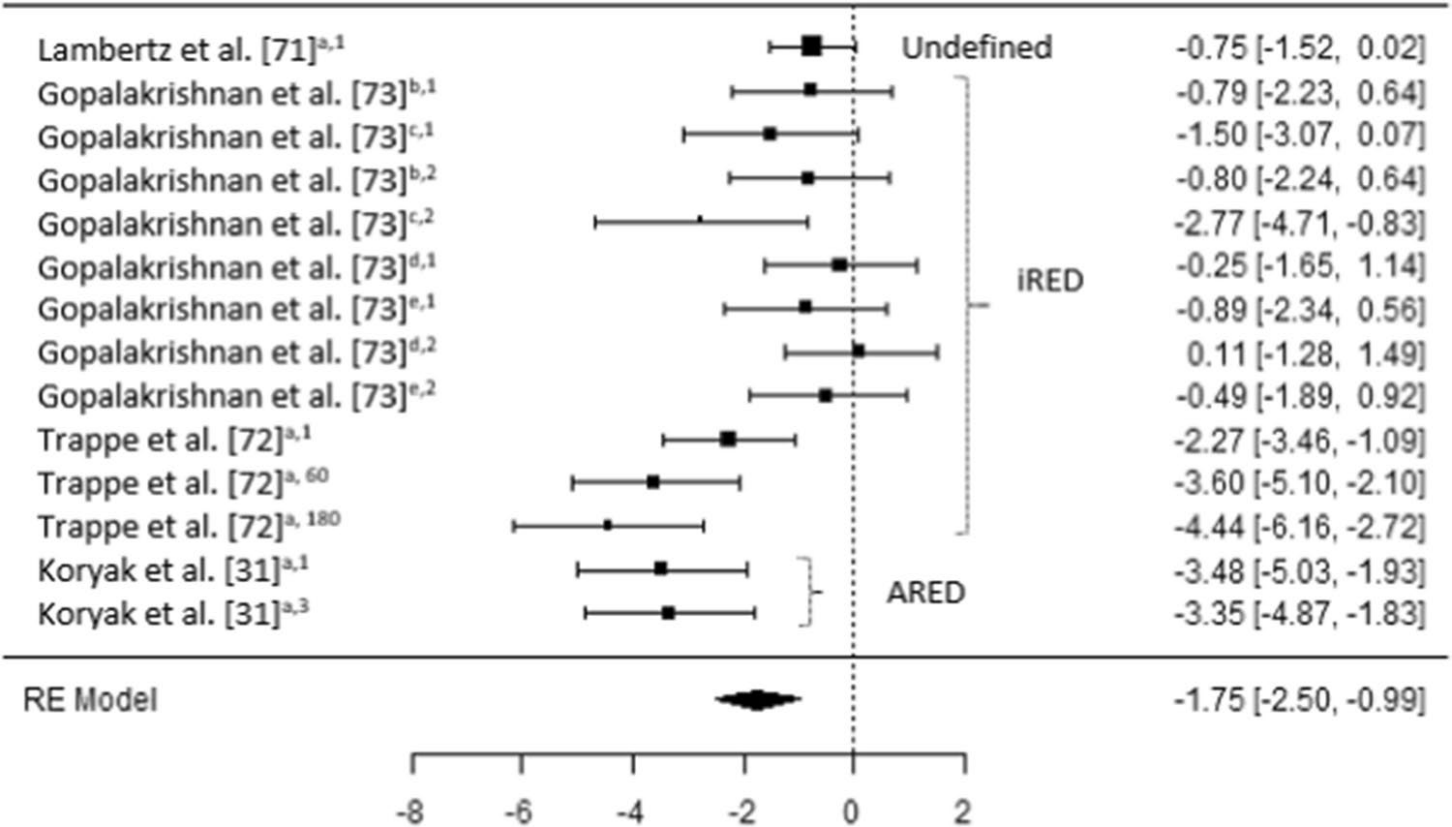
A comparison of changes (effect sizes and 95% confidence intervals) in muscle strength and endurance pre- to post-spaceflight. ^a^ = plantar flexion; ^b^ = knee extension; ^c^ = knee flexion; ^d^ = hip extension; ^e^ = hip flexion; ^1^ = maximum voluntary isometric contraction; ^2^ = muscular endurance, work; ^3^ = tetanic force production; ^60^ = isokinetic assessment at 60°.s^−1^; ^180^ = isokinetic assessment at 180°.s^−1^; iRED = interim resistive exercise device; ARED = advanced resistive exercise device. Values represent Hedge’s *g* effect size and 95% confidence intervals. Negative values (< 0.00) highlight a negative effect

**Fig. 6:**
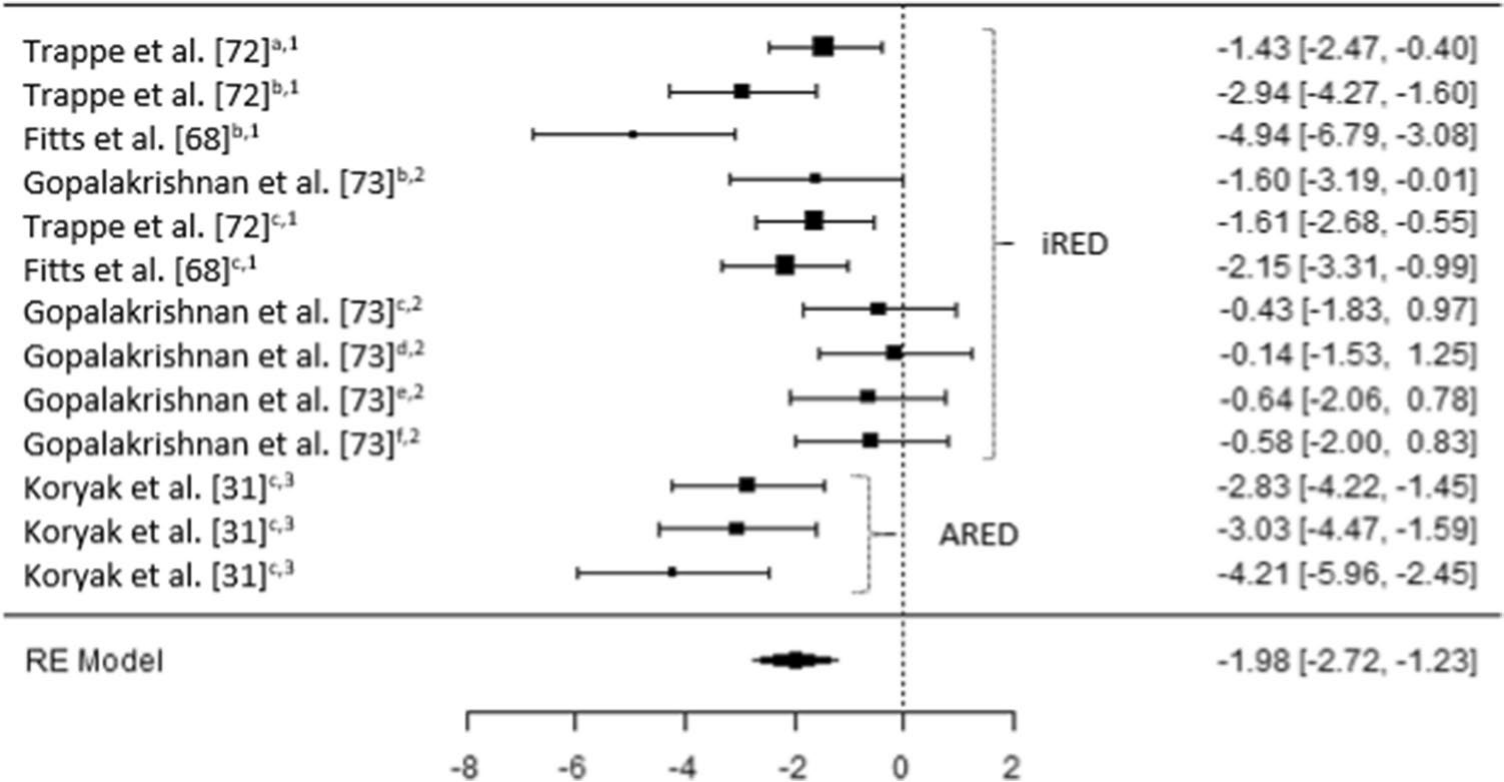
A comparison of changes (effect sizes and 95% confidence intervals) in leg muscle size pre- to post-spaceflight. ^a^ = combined calf (soleus and gastrocnemius); ^b^ = soleus; ^c^ = gastrocnemius; ^d^ = tibialis anterior; ^e^ = knee extensors; ^f^ = knee flexors; ^1^ = cross sectional area; ^2^ = volume; ^3^ = thickness; iRED = interim resistive exercise device; ARED = advanced resistive exercise device. Values represent Hedge’s *g* effect size and 95% confidence intervals. Negative values (< 0.00) highlight a negative effect

The results of the six meta-analyses were calculated, providing comparisons of the magnitudes of changes in BMD (femoral; trochanter; hip, pelvis and spine) lower limb force production, lower limb muscle size and spinal muscle size, pre- and post-spaceflight (Table 2). Spaceflight resulted in small reductions in BMD (Figs. 2, 3 and 4), although the magnitude of these reductions clearly decreases when the ARED exercise protocol is implemented and with the addition of bisphosphonates ingestion (Pre-ARED vs. ARED: Femur *g* = − 0.23 to − 0.92 vs. − 0.15 to 0.16; Trochanter *g* = − 0.41 to − 0.83 vs. − 0.02 to − 0.16; Lumbo-pelvic *g* = − 0.39 to -0.99 vs. − 0.04 to − 0.17). In contrast, lower limb muscle force production demonstrates a large decrease (*g* − 0.175 [− 0.250 to 0.99]) post-spaceflight (Fig. 5), with a similarly large decrease (*g* − 1.98 [− 2.72 to − 1.23]) in lower limb muscle size (Fig. 6), although only a small reduction (*g* = − 0.31 [− 0.59 to − 0.03]) in spinal muscle size is evident. In contrast to the changes in BMD, the current exercise regimes performed using the ARED do not appear to be an effective countermeasure to address the deleterious effects of µG on muscle function or muscle size.

**Table 2:** Meta-analytical statistics for musculoskeletal changes pre- to post-spaceflight

	Estimate	Z	*p*	95% CI	Tau^2^	I^2^ (%)	*P*	Fail safe N	*p*
Bone									
Femur	− 0.488	− 4.68	< 0.001	− 0.693 to − 0.284	< 0.001	0.00	0.980	93	< 0.001
Trochanter	− 0.530	− 4.38	< 0.001	− 0.767 to − 0.293	< 0.001	0.00	0.963	62	< 0.001
Lumbo-pelvic region	− 0.470	− 3.73	< 0.001	− 0.729 to − 0.227	< 0.001	0.00	0.964	43	< 0.001
Muscle									
Force production	− 1.75	− 4.52	< 0.001	− 2.504 to − 0.989	1.546	76.03	< 0.001	392	< 0.001
Lower body muscle size	− 1.98	− 5.17	< 0.001	− 2.724 to − 1.227	1.386	74.38	< 0.001	470	< 0.001
Spinal muscle size	− 0.306	− 2.14	0.033	− 0.586 to − 0.025	< 0.001	0.00	0.984	5	0.016

Z = z score, CI = confidence interval

The estimated variance (≤ − 0.306) for each of the meta-analyses was significant (*p* ≤ 0.033), in BMD (− 0.48 to − 0.53, *p* < 0.001), lower body force production (− 1.75, *p* < 0.001), lower body muscle size (− 1.98, *p* < 0.001) and spinal muscle size (− 0.306, *p* = 0.033).

### Study Quality and Bias Results

Heterogeneity of the completed meta-analyses was conducted revealing homogeneity for BMD and spinal muscle size (Tau^2^ < 0.001; *I*^2^ = 0.00%, *p* > 0.05), although a high level of heterogeneity was noted for lower body force production (Tau^2^ = 1.546; *I*^2^ = 76.03%, *p* < 0.001) and lower body muscle mass (Tau^2^ = 1.386; *I*^2^ = 74.38%, *p* < 0.001) (Table 2).

The PEDro scores for the studies included in the meta-analyses ranged from 3 to 6 (mean ± SD = 3.7 ± 1.2) (Table 3). Such scores indicate that the studies range from moderate to excellent in quality. The fail-safe *N* (using the Rosenthal method) identified that each meta-analysis was not subject to publication bias (p ≤ 0.016), with 43–93 “filed-away” studies required to provide null effects for changes in BMD, 392–470 “filed-away” studies required to provide null effects for changes in muscle force production and muscle mass, respectively. In contrast, only 5 “filed-away” studies were required to provide null effects for changes in spinal muscle size (Table 2).

**Table 3:** Modified Physiotherapy Evidence Database (PEDro) scale for included studies

Study	1	2	3	4	5	6	7	8	9	10	11
Lambertz et al. [71]	✓	x		x				✓	✓	x	x
Lang et al. [60]	✓	x		x				✓	✓	x	x
Lang et al. [61]	✓	x		x				✓	✓	x	x
Trappe et al. [72]	✓	x		x				✓	✓	x	x
Fitts et al. [68]	✓	x		x				✓	✓	x	x
Gopalakrishnan et al. [73]	✓	x		x				✓	✓	x	✓
Smith et al. [54]	✓	x		✓				✓	✓	✓	✓
LeBlanc et al. [53]	✓	x		✓				✓	✓	✓	✓
Burkhart et al. [78]	✓	x		x				✓	✓	x	x
Koryak et al. [31]	✓	x		x				✓	✓	x	✓
McNamara et al. [79]	✓	x		x				✓	✓	x	x
	= Excluded as these criteria are not feasible in such studies										

1: Eligibility criteria were specified. 2: Subjects were randomly allocated to groups. 3: Allocation was concealed. 4: The groups were similar at baseline, regarding the most important variables. 5: There was blinding of all subjects. 6: There was blinding of subjects and therapists. 7: There was blinding of assessors who measured at least one key outcome. 8: Measures of at least one key outcome were obtained from > 85% of subject initially allocated to groups. 9: All subjects for whom outcome measures were available received the treatment or control condition as allocated or, where this was not the case, data for at least one key outcome was analysed by “intention to treat”. 10: The results of between-group statistical comparisons are reported for at least one key outcome. 11: The study provides both point measures and measures of variability for at least one key outcome

## Discussion

The purpose of this systematic review and meta-analysis was to determine the effects of long-term (≥ 30 days) spaceflight on skeletal health (BMD) and muscle function (force production and muscle mass), while evaluating the effects of existing countermeasures used to minimize the deterioration of the musculoskeletal systems during spaceflight. It is evident that small reductions in BMD occur as a result of long-duration spaceflight, although the magnitude of these reductions clearly decreases when the ARED exercise protocol is implemented and with the addition of bisphosphate ingestion. In contrast, there are large decreases in lower limb muscle force production after long-duration spaceflight, with a similarly large decrease in lower limb muscle size, irrespective of the exercise countermeasures; although only a small reduction in spinal muscle mass is evident.

### Changes in Bone Mineral Density

Irrespective of the bone (e.g., femur, pelvis, spine) or bone region (e.g., integral, cortical, trabecular) prior to the implementation of the ARED and associated exercise protocols, small to moderate reductions in BMD were evident post long-duration spaceflight (Hedges *g* = − 0.23 [95% CI − 0.98 to 0.51] to − 0.99 [95% CI − 2.03 to 0.05]) (Figs. 2, 3 and 4). It is worth noting that the studies by Lang et al. [60, 61] do not clearly state if the iRED device and exercise protocols were implemented by the crew members, or whether the treadmill was the primary mode of exercise (Table 4). Lang et al. [60] did, however, report that the greatest rates of mineral loss occurred in trabecular bone (2.2–2.7% per month) compared to cortical bone (1.6–1.7% per month), highlighting the importance of monitoring specific bone regions. Averaging total bone mass, as with dual X-ray absorptiometry (DXA), has previously been reported to obscure changes in trabecular mass, due to the lower decreases in the highly dense cortical bone [62, 63]. Additionally, in 2012, experts at the NASA Bone Summit highlighted that DXA measures are unable to capture the full effects of spaceflight on skeletal health with recommendations to include quantitative computed tomography (QCT) derived finite element (FE) models [64]. Such findings are in line with those reported in a previous review where 92% of astronauts and cosmonauts (*n* = 60) on long-duration missions aboard the Mir space station and ISS demonstrated BMD decreases of ≥ 5% with 43% demonstrating decreases ≥ 10% in at least one site [3], prior to the use of the ARED.

It has been suggested that the impact forces associated with treadmill running may beneficially attenuate reductions in BMD in the calcaneus, femur and spine [60]. It should be remembered, however, that it is a combination of the magnitude, rate and frequency of strain which appear to be the stimulus for skeletal adaptation or maladaptation [65]. It is evident from the results of current meta-analysis that either the magnitude of strain, rate of strain, frequency of strain or a combination of all three stimuli appears to have been insufficient prior to the implementation of the ARED exercise protocol (Figs. 2, 3 and 4). As the frequency of the exercise protocols and the recommendations for treadmill running associated with the iRED and ARED are the same (Table 1), it is likely that the magnitude of loading between the devices (maximum load: iRED = 136 kg vs. ARED = 272 kg) is responsible for the differences in the changes in BMD, with the ARED providing the most beneficial stimulus, with only trivial changes in BMD (Hedges *g* = − 0.17 [95% CI −1.41 to 1.07] to 0.16 [95% CI − 0.89 to 1.21]) (Figs. 2, 3 and 4), especially when combined with bisphosphonate ingestion [53]. While it may appear that both of these loads are substantial, it is worth highlighting that astronauts do not have to account for their mass during exercises such as squats, deadlifts and heel raises, as they would on Earth, as a result of reducing the system mass (external load + body mass) associated with the exercises while in µG compared to when on Earth [66]. With a mean astronaut body mass of ~ 80.5 ± 11.7 kg [38] the iRED permits a maximum equivalent external load (compared to Earth) of ~ 55.5 kg (68.9% of typical astronaut body mass), whereas the ARED results in a maximum equivalent external load of ~ 191.5 kg (236.6% of typical astronaut body mass), during lower body exercises. However, the addition of body mass to the external load applied from the ARED device has been questioned, in terms of whether the lumbo-pelvic musculature and the axial skeleton could safely tolerate the loads [66, 67], although this may be addressed with the adoption of a belt-squat style squat to provide the legs with a sufficient stimulus [67]. It is also worth noting that the treadmill upgrade in 2009 and the replacement of TEVIS with T2 provided an increased potential to run at higher speeds (12 mph vs. 10 mph); however, based on published training logs, it appears that this capacity is yet to be regularly utilized [68]. A balance between appropriate musculoskeletal loading to offset the detrimental effects of µG, and risk of injury is essential to ensure that mission objectives are not compromised by the ability of the crewmember to complete essential tasks; however, progressive strength training resulting in an increased level of strength has been shown to reduce the risk of musculoskeletal injury in athletic populations [69, 70].

### Changes in Muscle Function and Muscle Size

**Fig. 7:**
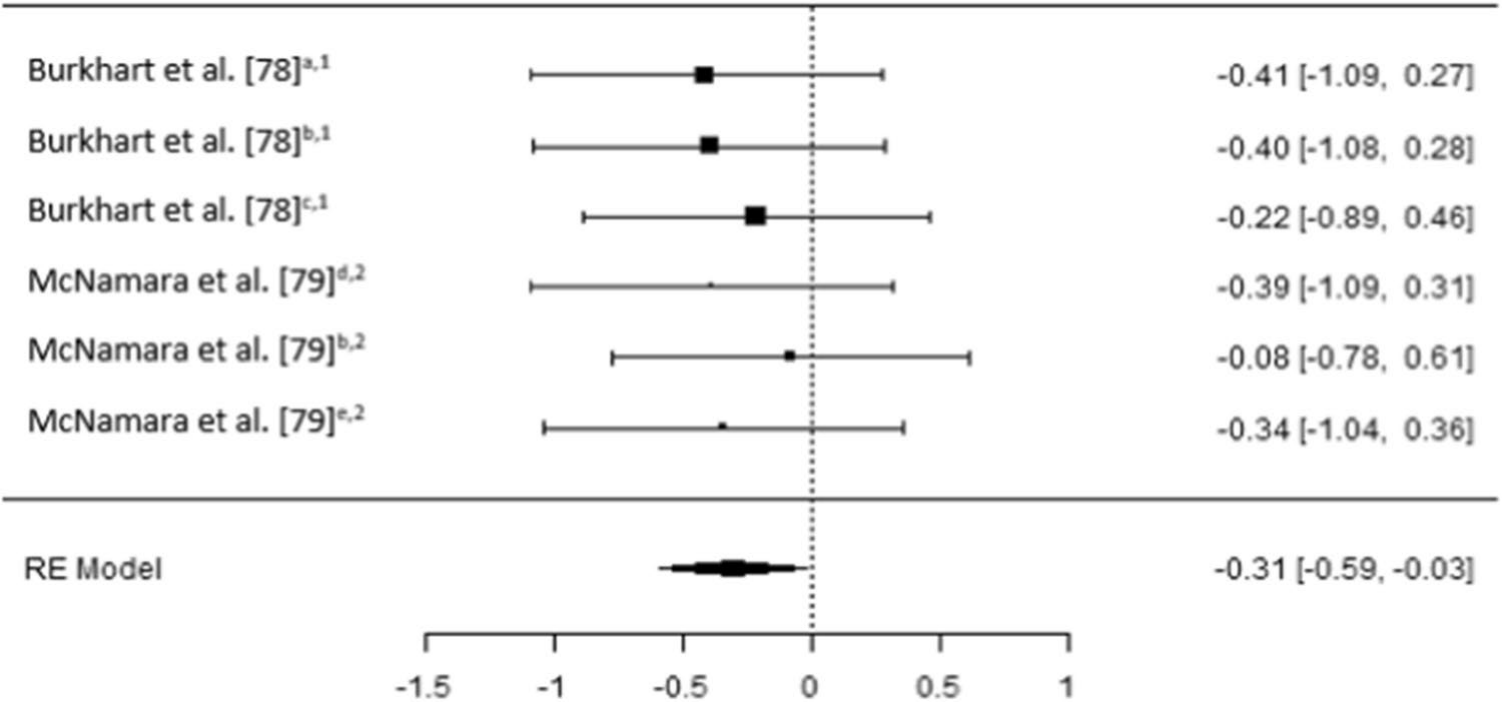
A comparison of changes (effect sizes and 95% confidence intervals) in spinal muscle size pre- to post-spaceflight (interim resistive exercise device intervention). ^a^ = multifidus; ^b^ = erector spinae; ^c^ = psoas; ^d^ = paraspinal muscles; ^e^ = quadratus lumborum; ^1^ = cross sectional area; ^2^ = volume. Values represent Hedge’s *g* effect size and 95% confidence intervals. Negative values (< 0.00) highlight a negative effect

In contrast to the moderate to trivial changes in BMD, large decreases (Hedges *g* = − 1.75 [95% CI − 2.50 to − 0.99]) in lower limb muscle force production occur after long-duration spaceflight irrespective of the countermeasures used (Fig. 5). Additionally, a similarly large decrease (Hedges *g* = − 1.98 [95% CI − 2.72 to − 1.23]) occurs in lower limb muscle size (Fig. 6); although only a small reduction (Hedges *g* = − 0.31 [95% CI − 0.59 to − 0.03]) occurs in spinal muscle size (Fig. 7).

The magnitude of the decrease in force production appears to differ somewhat, based on the muscles assessed and the speed of the assessment method (e.g., 60° s^−1^; Fig. 5). Overall, lower limb postural muscles with a higher percentage of slow twitch fibers (e.g., plantar flexors) demonstrate a larger decline than muscles with a higher percentage of fast twitch fibers; however, no researchers appear to have directly compared such differences between muscles, with Lamberts et al. [71], Trappe et al. [72] and Koryak et al. [31] assessing the plantar flexors, while Gopalakrishnan et al. [73] assessed the knee and hip flexors and extensors (Table 5). In the plantar flexors, as movement velocity increases (e.g., MVIC to angular velocities of 60° s^−1^ and 180° s^−1^), the magnitude of reduction in force increases [72], in line with observations from short-duration spaceflight [25]. These greater decreases in force at higher velocities are likely a product of neurological [4, 33] and architectural changes [31], but may also be attributable to muscle fiber type shifts observed in bedrest studies [74, 75]. Similar findings have also been observed during short-duration unloading of the plantar flexors on earth [76] and during spaceflight [25–29, 77]. Interestingly, Gopalakrishnan et al. [73] demonstrated small decreases in hip extensor MVIC (Hedges *g* = − 0.25 [95% CI − 1.65 to 1.14]), along with a trivial increase (Hedges *g* = 0.11 [95% CI − 1.28 to 1.49]) in work capacity; in contrast, knee flexor work capacity demonstrated very large (Hedges *g* = − 2.77 [95% CI − 4.71 to 0.83]) decreases (Fig. 5), although clear variation is evident between the four crew members, based on the 95% CI. It is likely that such differences in the change in function of different muscles are attributable to the exercises performed using the iRED, with numerous exercises relying on hip extension (deadlift, squat, Romanian deadlift) but none focusing on knee flexion. The regular performance of exercises which result in a large demands on the paraspinal muscles may also explain why the decreases in the size of these muscle is small (Fig. 7) [78, 79].

Laughlin et al. [49] reported a trivial (Hedges *g* = − 0.01 [95% CI − 0.28 to 0.21] 2.5 ± 7.2%) decrease in leg press strength, with no meaningful changes (< 1.0%) in pull-up or bench press performance. However, they did note that the variance in leg press performance in their laboratory was 5–10% and, therefore, was unlikely to be sensitive enough to detect small changes in performance. The potential differences in the regional effects on muscle strength are in line with previous observations, where weight bearing muscle tends to exhibit greater losses in size and strength than non-weight bearing muscles [19, 80]. The results of this study were not included in the meta-analyses, due to the subjects using a combination of the iRED and ARED devices.

In line with observations from short-duration spaceflight [27–29, 34, 35, 77, 81] and similar to the changes in force production after long-duration spaceflight, the greatest decreases in muscle mass are observed in the lower leg muscles compared to the larger, more fast twitch, thigh and hip musculature (Fig. 6). However, as with force production, each of these muscles has not been effectively compared in a single study and, therefore, other confounding variables [e.g., mission duration (Table 6)] [3, 4, 11], adherence to exercise protocols [40], energy balance [5, 9], and baseline physical capacity [41]) may have influenced such changes and, therefore, the differences observed between studies.

Interestingly, the introduction of the ARED and associated exercise protocol does not appear to have had a positive influence on the reductions in force production or muscle mass after long-duration spaceflight (Figs. 5 and 6), unlike its effect on BMD (Figs. 2, 3 and 4). In fact, the magnitude of decrease in MVIC and muscle mass appears to be greater when the ARED protocol was used (Hedges *g* = − 2.83 [95% CI − 4.22 to − 1.45] to − 4.21 [95% CI − 5.96 to − 2.45]) [31] compared to when the iRED was used (Hedges *g* = − 0.14 [95% CI − 1.53 to 1.25] to − 2.15 [95% CI − 3.31 to − 0.99]). This was true for most muscles studied, except for soleus CSA in the studies by Trappe et al. [72] and Fitts et al. [68]. These results are interesting, as when the ARED protocol was compared to free weight exercise on Earth, comparable improvements in BMD, strength, power and muscle size, albeit in previously untrained subjects, were observed [82]. Additionally, Petersen et al. [38] report a higher number of training sessions completed after the installation of ARED compared to iRED, yet this increased frequency does not appear to have prevented the deleterious effects of µG. Interestingly, Gopalakrishnan et al. [73] also reported large decreases in soleus muscle volume (Hedges *g* = − 1.60 [95% CI − 3.19 to − 0.01]; however, these were much smaller in magnitude compared to those reported by Trappe et al. [72] and Fitts et al. [68], which may be attributable to the higher intensity range (3.09–7.15 mph) of treadmill exercise used by crew members in the former study, compared to the lower intensity range (2.1–5.5 mph) in the latter two studies. It is worth noting that these sessions are less demanding than if performed on earth, as only ~ 80% body mass is applied when using the treadmill on the ISS (potentially reducing typical ground reaction forces from around 2400 N to around 1900 N [based on typical, normal gravity condition, running forces and typical astronaut body mass of 80.5 kg]).

It is currently unclear why the adoption of the ARED protocol does not appear to result in improvements in the maintenance of muscle function and mass, similar to the improved maintenance of BMD. There are, however, numerous possible contributing factors, including the roles of adherence to the recommended exercise protocols [38, 40], which can be impacted by mission demands [38, 42] and the functional status of the exercise equipment [42], energy balance [5, 9], pre-spaceflight training status [41, 83] or the influence of concurrent training [84, 85]. Concurrent training has been shown to adversely affect muscle function and mass in military [86] and sporting populations due to an interference effect from aerobic exercise [87–90]. Recently, Jones et al. [85] explored the potential impact of concurrent training in µG, concluding that the high frequency, moderate intensity and total volume of aerobic training may negate the positive effects of resistance exercise on the maintenance of muscle mass and function.

As already mentioned, while the loads permitted via the iRED and ARED devices may appear substantial, this does not account for the negated mass of the astronaut during exercises such as squats, deadlifts and heel raises, compared to exercising on Earth, resulting in reduced system mass. More importantly, when considering the loads used during exercises such as the squat, deadlift and heel raises, it appears that the median loads used are not substantially greater than the mean body mass of the astronauts [38] and are therefore likely to be far too conservative to maintain muscle mass or function during prolonged periods of µG. Such low loads have only been shown to be effective at increasing muscle mass and strength in untrained individuals, if repetitions are performed to momentary muscle failure, although these adaptations appear to be predominant in slow twitch muscle fibers [91]. In addition, higher-load resistance training results in greater improvements in strength compared to low-load training [50], with Kozlovskaya and Grigoriev [83] previously reporting an association between the frequency and intensity of exercise and the maintenance of musculo-skeletal health in cosmonauts.

### Current Training Prescription

During spaceflight on the ISS, exercise duration is progressively increased over the first 3 weeks, from an initial 60 min bout up to sessions lasting ~ 2.5 h per day [38], which also includes time for dressing, set-up of equipment and cleanup. The use of such long-duration, high-frequency (4–6 days/week) exercise (Table 1) has been questioned by Laurens et al. [10] due to issues associated with the maintenance of energy balance and therefore body mass and composition. Interestingly, twenty years ago, Stein [5] suggested that ‘an inappropriate inflight exercise regimen is counterproductive’, especially if this contributes to a chronic energy deficit. Importantly, astronauts have been reported to consume hypocaloric diets when in space, even though sufficient food is available [6, 8, 9], which would exacerbate weight loss when high volumes of exercise are being performed, thereby jeopardizing the success of long-term missions [5, 92]. While beyond the scope of this review it is also important to be mindful of the maintenance of protein intake, to minimize muscle atrophy [93–95], especially in an environment where food sources are limited and may degrade more rapidly due to higher levels of radiation. Based on the available data Laurens et al. [10] predict that a mission to Mars would currently result in serious health implications attributed to ~ 15% loss in body mass, although this prediction does not appear to take into account the gravity (0.38 G) on the surface of Mars. In contrast, in one study each additional weekly iRED session was predicted to result in a 2.4% improvement in lumbopelvic muscle volume retention (*R*^2^ = 0.72) [79]; however, the results of this meta-analyses highlight minimal reduction in lumbo-pelvic muscle mass during prolonged spaceflight (Fig. 7) and therefore, this is of less concern than the loss of mass and function in the lower limbs.

In-flight exercise is divided into three phases; Phase 1 lasts ~ 20 days commencing with relatively low intensities (50–60% of pre-spaceflight capacity), to provide an adaptive phase to µG. Phase 2 results in a progressive increase in resistance exercise intensity of 3–5% per week, although this is less structured for aerobic exercise (e.g., treadmill or cycle ergometer), based on crewmember performance, but with an aim of achieving ~ 80% of the individual’s capacity (Table 1). Phase 3 (preparation for Re-entry) lasts 3–4 weeks with increased focus on resistance exercise and treadmill running at high intensities in preparation for terrestrial loading [38]. Treadmill loading is increased progressively through the astronauts’ time on the ISS, with the implementation of three phases: Phase 1—using loads of ~ 50% body weight (first 20 days), Phase 2—loads are increased to 70–80% body weight (depending on individual tolerance and comfort) (Table 1), Phase 3—increased loads if tolerable [38]. During missions astronaut strength conditioning and rehabilitation (ASCR) personnel individualize training based on the weekly training logs of crewmembers [40]. However, based on training log data, individual compliance to such exercise recommendations is highly variable [42, 72, 73], with generally low-intensity (2.1—5.5 mph [walking—jogging]) treadmill exercise and low-load resistance exercise (12–20 repetitions per set) reported [72]. The large decreases in muscular force production and muscle size during long-duration spaceflight are unsurprising, as these practices are far from the existing recommendations for terrestrial strength development (3–5 sets, ≤ 6 repetitions, with loads of ≥ 85% one repetition maximum [1RM]) and hypertrophy 3–5 sets, 8–12 repetitions, with loads of 67–85% 1RM) [96]. While lower resistance training loads have been shown to result in hypertrophy [50, 97], it is clear that greater increase in muscle mass and strength are associated with loads > 60% 1RM [50, 97, 98]. When higher loads are used (7 sets of 3 repetitions, ~ 90% 1RM) compared to moderate loads (3 sets or 8–12 repetitions, ~ 70% 1RM) and the volume is equated, similar increases in muscle size occur, but the higher-load training results in greater increases in strength [98]. In addition, more frequent training appears to result in greater increases in strength [99]; however, this appears to be explained by the increase in weekly training volume rather than frequency [100]. Interestingly, improved musculoskeletal health of cosmonauts, after prolonged spaceflight, has been reported in those who undertake higher-intensity and more frequent exercise [83], similar to previous observations [41], with crewmembers who adhere more closely to the existing exercise recommendations demonstrating smaller decrements in musculoskeltal health [42].

On the ISS, recommendations for resistance training consist of 3–5 sets of each exercise, using daily rotations of 6, 8, 15 repetitions, including lower body exercises, such as squats, deadlifts and heel raises [38, 68, 72, 73] (Table 1); however, most training logs report repetition ranges of ≥ 12 repetitions per set [68, 72, 73]. Additionally, as already highlighted, it is evident that the loads used are rather limited (only slightly greater than body mass during exercises which, on Earth, would include substantial external load in addition to body mass) [38] and are likely a contributing factor to the progressive decrease in muscle size and muscle function. Low loading during bilateral squats, deadlifts and heal raises have been highlighted using in shoe force assessments, with only unilateral squats and unilateral heel raises resulting in forces greater than body weight [73], which is unsurprising based on the loads reported in the exercise logs of the crewmembers [68, 72, 73]. Researchers have previously concluded that the moderate resistance exercise loads used while on the ISS may be insufficient in the maintenance of muscle mass and strength [68, 72], with Kozlovska et al. [42] recommending higher-intensity exercise. Ideally, moderate loads (8–12 RM [~ 70% 1RM]) performed for 8–12 repetitions, to momentary muscle failure, have been shown to be most effective for combined hypertrophic and strength adaptations [50, 91, 97] and therefore, most likely ideal for maintenance of muscle mass.

### Concurrent Training

Continuous, moderate-intensity exercise, especially in high volumes, in close proximity to resistance training has been reported to compromise adaptations to resistance training via inhibition of the mammalian target of rapamycin (mTOR) pathway, due to elevated adenosine monophosphate-activated protein kinase (AMPK) [87, 101] originally referred to as the *interference effect* [102]. As a result of this interference effect, adaptations primarily in response to resistance training (e.g., hypertrophy, increased force production) are reduced, while adaptations to the cardiovascular system do not appear to be meaningfully affected [84, 85, 87]. Interestingly, Trappe et al. [72] reported that during a 6-month period on the ISS astronauts performed moderate-intensity aerobic exercise 5 days per week while resistance training ranged from 3 to 6 days per week. Greater emphasis on aerobic training highlights the potential for an interference effect, which may minimize the effect of the resistance exercise as an effective countermeasure, especially in light of the reported low-intensity and long-duration aerobic exercise. Fitts et al. [68] also concluded that the emphasis of aerobic exercise appears to prevent muscle mass and function loss, although they did identify that atrophy was reduced in individuals who performed < 100 min/week of treadmill exercise compared to those who performed > 200 min/week, which may have been worsened by an energy intake ~ 20% lower than the predicted requirement of the crewmembers [72]. To reduce the potential deleterious effect of concurrent training, it may be advantageous to reduce the weekly duration of low–moderate-intensity aerobic exercise and increase the intensity or replace some sessions with high-intensity interval training.

Jones et al. [85] have recently explored the potential deleterious effects of concurrent training during spaceflight concluding that resistance exercise should not be performed in close proximity (ideally > 4 h) to aerobic exercise, but that if this cannot be avoided resistance exercise should precede aerobic exercise, with eccentric training methods included, if feasible, to increase load while decreasing metabolic cost. Unfortunately, based on the recent observations of cosmonauts, it is unlikely that crewmembers would be able to, or choose to, exercise twice per day, likely due to the time required to set up equipment, dress and clean up [42, 83]. Additionally, Jones et al. [85] also recommended high-intensity intermittent training (HIIT) in place of steady-state aerobic exercise, although they suggested avoiding treadmill running as this was previously reported to have a greater interference effect than cycling [84]; however, during spaceflight, the additional loading on the skeletal system, associated with the impact from running, is likely essential to maintaining BMD. Results of a recent meta-analysis indicate that the interference effect from running can be negated if HIIT practices are implemented [90], with such protocols likely to result in higher ground reaction forces during the high-intensity periods, which may be advantageous to the maintenance of BMD. On the ISS maximal treadmill speeds of 20 km/h [38] should permit a sufficient intensity for HIIT training.

### Limitations, Recommendations and Areas of Future Research

Due to the number of subjects per study and the range of mission durations across crew members within studies, it was not possible to determine the effect of increased mission duration on changes in musculoskeletal health; however, musculoskeletal deterioration has been shown to progress with an increase in mission duration [3, 4, 11]. In addition, due to the low number of studies which included the same countermeasures, especially ARED, it was not possible to include exercise protocol as a moderator, although the trends do appear quite clear (Figs. 2, 3 and 4, 5, 6 and 7), as already discussed. It is worth noting that when crewmembers do adhere closely to the existing exercise recommendations, they demonstrate smaller decrements in musculoskeletal health [42]; therefore, individualization of training may benefit from a less conservative, higher-load approach from ASCR personnel. It also appears as though the exclusion of body mass during resistive exercise on the ISS is not effectively considered, which likely results in the higher repetitions and lower loads than those currently recommended while onboard the ISS [38]. While, the addition of body mass to the external load applied from the ARED device has been questioned, in relation to the strength of the lumbo-pelvic musculature and the axial skeleton to safely tolerate the loads [66, 67], the adoption of a belt-squat would provide the legs with a sufficient stimulus [67] and can be easily achieved with a flywheel device if the ARED cannot accommodate this exercise or the required level of resistance. Such flywheel devices have been used in studies where bedrest is used as an analogue for µG, with the results of such studies highlighting that such countermeasures can negate the detrimental effects of simulated µG in, terms of force production, muscle mass and fiber type shifts [74, 75, 103, 104] and may offset the interference effects of concurrent training [105]. It is not clear whether these beneficial effects of such countermeasures are a result of the higher loads and lower repetitions used, when compared to actual exercise reported by ISS crewmembers, or due to the differences in simulated and actual µG [2, 5, 46, 47].

Based on the findings of this review, it is recommended that higher-load resistance training, appropriate for the use of daily rotations of 6, 8, 15 repetitions, in line with the current guidelines for resistive exercise on the ISS [38], are utilized, ensuring a sufficient load to minimize the reductions in muscle strength and size, in line with terrestrial guidelines [96] and previous recommendations [83]. Additionally, to minimize the effect of concurrent training, and potentially reduce energy expenditure, replacing some of the prolonged, low–moderate-intensity aerobic training sessions with HIIT may be advantageous [66, 85] and have previously been recommended [42, 83]. If some of the HIIT sessions are treadmill based, utilizing the higher running speeds available on T2, this may further mitigate the reductions in BMD, due to the associated impact forces, while also providing a greater stimulus of the calf musculature, which appears to demonstrate the greatest declines in muscular force and muscle size. Further exploration of the effects of high-load (~ 90% 1RM) strength training on both muscle mass and force production should also be explored, as terrestrial results demonstrate positive adaptations for both [98]. Finally, the exercise protocols using flywheel devices, that have been shown to be very beneficial in simulated µG studies should be evaluated in actual µG [74, 75, 103]. Some of these flywheel devices permit an array of appropriate exercises to be performed, and they are small and lightweight, which may be advantageous during interplanetary travel where space and payloads will likely be limited.

In the future, researchers should determine the effect of higher-load resistance training and HIIT, while on the ISS, to take advantage of the loading capacity of the ARED and the speed of the T2 treadmill, which based on current training logs are yet to be fully exploited. Prior to evaluating such training protocols during spaceflight, it would be advantageous to determine the effects of increased load during resistance training and increased intensity of aerobic exercise, including HIIT, throughout the pre-spaceflight preparation on Earth. The effect of increased pre-flight strength and aerobic capacity or changes during spaceflight should also be instigated, as higher pre-flight levels may off-set reductions during missions [41]. Additionally, Jones et al. [85] have recommended investigating the potential use of eccentric training, which could be used as a supplementary method to the current resistance training practices, once the effects of higher-load resistance training has been established. Improved reporting in training logs would also be advantageous and may be facilitated by advances in wearable technology, potentially synchronized with the ARED, treadmill and cycle ergometer, to both monitor and record the actual demands of training performed.

As baseline aerobic capacity has been shown to positively affect aerobic exercise intensity in space, supporting the maintenance of aerobic capacity [41], it would be useful to determine if higher baseline BMD, muscle strength and muscle mass positively influence post-spaceflight outcomes. Data may already be available to provide a retrospective analysis to determine such associations, while future pre-spaceflight physical conditioning may need to emphasize the development of muscle strength and muscle mass, to offset any deterioration associated with long-duration spaceflight.

## Conclusions

Current exercise countermeasures, incorporating the ARED and associated exercise protocols, and the way in which they are currently adopted by crewmembers, appear to minimize the reductions in BMD associated with long-duration spaceflight, especially when combined with bisphosphonate ingestion [53]. In contrast, the way in which these countermeasures are adopted by crewmembers are insufficient to maintain muscle function and muscle mass, likely attributed to insufficient loading strategies, along with the potential interference effects of concurrent training. For successful interplanetary travel, which minimizes the detrimental effects of µG on the muscular system, the adoption of improved countermeasures is essential.

## Data Availability

The data within this study are secondary data and available through the relevant articles referenced throughout. All statistical analyses were carried out using Jamovi [52], an open source software that is freely available.
